# Outcomes of internal biliary diversion using cholecystocolostomy for patients with severe Alagille syndrome

**DOI:** 10.1093/jscr/rjac307

**Published:** 2022-07-04

**Authors:** Jonathan M Durgin, Robert Crum, Heung Bae Kim, Alex G Cuenca

**Affiliations:** Department of Surgery, Boston Children’s Hospital, Boston, MA, USA; Department of Surgery, Boston Children’s Hospital, Boston, MA, USA; Department of Surgery, Boston Children’s Hospital, Boston, MA, USA; Department of Surgery, Boston Children’s Hospital, Boston, MA, USA

**Keywords:** Alagille syndrome, biliary diversion, cholecystocolostomy, cholestasis

## Abstract

Alagille syndrome (AGS) is a disorder that leads to increased serum cholesterol and bile acids, which can result in debilitating xanthomas and pruritus. External biliary drainage and transplantation are effective treatments for AGS. Internal biliary diversion with Roux-en-Y cholecystocolostomy has been described for other biliary conditions, but not AGS. Three patients with severe pruritus due to AGS underwent Roux-en-Y cholecystocolostomy for internal biliary drainage. Retrospective analysis compared preoperative and post-operative lab values and symptom scores (0, none–4, severe). Three patients underwent cholecystocolostomy. All patients had at least three diagnostic criteria for AGS. Mean preoperative pruritus score was 3.33 (range, 2–4) and mean post-operative score was 1. Mean preoperative xanthoma score was 1.33 (range, 0–4) and post-operative score was 1 at 2-month follow-up. Roux-en-Y cholecystocolostomy can be considered for AGS, which is refractory to medical management. This procedure accomplishes internal biliary diversion without significant physiologic derangements.

## INTRODUCTION

Alagille syndrome (AGS) is a rare disorder of the biliary system that can lead to progressive liver failure. AGS is associated with mutations in the *JAG1* gene, which is believed to affect the Notch signaling pathway that helps to direct cell fate in early embryonal development [1]. In addition to the biliary anomalies, AGS is also characterized by dysmorphic facies, congenital heart defects, most commonly pulmonary artery stenosis, vertebral defects and anterior chamber defects in the eye [2]. A paucity of bile ducts causes ineffective bile excretion and resulting cholestasis. Patients may experience severe debilitating symptoms due to the accumulation of cholesterol and bile acids. These include debilitating xanthomas and pruritus which can result in self-mutilation [3]. These symptoms may be refractory to medical therapy, leading to the consideration of surgical intervention.

There are several surgical options for AGS patients with symptoms refractory to medical treatment. External biliary drainage with successful improvement in symptoms has been described but requires maintenance of the external collection system [4–7]. External collection bags need to be changed, can leak and often produce an undesirable cosmetic result for patients. Ileal exclusion has also been shown to be successful. This procedure eliminates the need for external drainage and is reversible in cases of malnutrition or dehydration [8].

Another option may be cholecystocolostomy. Diao *et al*. described the use of a laparoscopic cholecystocolostomy for the treatment of progressive familial intrahepatic cholestasis (PFIC) in a series of 20 children. This technique was 85% effective, defined as normalization of bile acids at 12 months and had no major morbidity or mortality, including cholangitis [9]. To our knowledge, this technique has not been reported in patients with AGS. Herein, we report the outcomes following the creation of a cholecystocolostomy in three AGS patients with symptoms refractory to medical management.

## CASE SERIES

Three patients with AGS were identified via retrospective review of records between January 2001 and December 2012. They were referred to the pediatric surgery service for the evaluation of cholestasis refractory to medical therapy. A multidisciplinary decision was made to proceed with cholecystocolostomy, and informed consent was obtained for the purpose of internal biliary drainage.

### Surgical procedure

Two patients underwent a laparoscopic-assisted technique, and one patient underwent an open procedure. The laparoscopic technique was accomplished by inserting a laparoscope through the umbilicus. An additional 5-mm port was placed suprapubically and another was placed in the right upper quadrant. The colon was mobilized from the cecum to the sigmoid using a sealing device. This allowed for a tension-free Roux limb to reach the right upper quadrant. The right upper quadrant incision was enlarged to allow for the creation of anastomoses. The transverse colon, just proximal to the splenic flexure, was divided using a single fire of a stapler. A hand-sewn colocolostomy was created between the proximal colon and the descending colon just distal to the splenic flexure. The gallbladder was then incised with electrocautery. A single layer running PDS suture was used to create the Roux-en-Y cholecystocolostomy. The open technique was performed in a similar fashion with the exception that all portions of the case were performed through a single, small right upper quadrant incision.

### Analysis

After Institutional Review Board approval, patient records were reviewed for the collection of pertinent data, including demographics, operative details and lab values. Pruritus severity and xanthoma burden were scored by the authors from the caregiver and physician description of symptoms and physical examinations. Pruritus was scored according to the system used by Whitington: 0, none; 1, rubbing or mild scratching when undistracted; 2, active scratching without evident skin abrasions; 3, abrasions evident and 4, cutaneous mutilation, hemorrhage and scarring evident [4]. Xanthoma burden was scored according to a similar system: 0, none; 1, minimal, <20 scattered individual lesions; 2, moderate, >20 lesions not interfering with or limiting activities; 3, disfiguring, large numbers of lesions causing distortion of the face or extremities and 4, disabling, large numbers of lesions interfering with walking or hand use because of excess size and number [5].

## RESULTS

Two patients underwent laparoscopic-assisted cholecystocolostomy for the purpose of internal biliary drainage. One patient underwent an open cholecystocolostomy through a single right upper quadrant incision. The mean age was 7.5 years old at the time of operation. Patient demographic data, including criteria for AGS, medical therapies and liver disease, are available in Table 1. One patient was found to have a mutation in the *JAG1* gene. Two patients were found to have fibrosis on preoperative liver biopsy. One patient was followed for 12 days and resumed care with their primary providers due to geographic limitations. Of the patients in the series, two patients had Grade 4 pruritus, and one had Grade 2. Two patients had no preoperative xanthomas, one patient had Grade 4 xanthomas leading to disfigurement and disability (Table 2). The roux limbs measured 9–25 cm. Mean time to resumption of a full diet was 5.3 days (range, 4–7). Total length of hospital stay was an average of 7.6 days (range, 5–10).

**Table 1:** Demographic data and pertinent history of 3 patients with AGS undergoing cholecystocolostomy

Patient	Sex	Age at time of operation	Diagnostic criteria for AGS	Medical therapies	Liver disease
1	F	12 years 8 months	h, c, f	u, r, h	Unknown
2	M	1 years 3 months	h, c, v, g	u, r, n, h	Fibrosis
3	F	2 years 4 months	h, c, r	u, r, n, c	Fibrosis

Diagnostic criteria: h, cholestatic liver disease or bile duct paucity on biopsy; c, peripheral pulmonic stenosis or other cardiac lesions; v, vertebral anomalies; r, renal anomalies; g, genetic confirmation; f, characteristic facies. Previous therapies: u, ursodeoxycholic acid; r, rifampin; c, bile acid-binding resins; h, antihistamines.

**Table 2:** Preoperative and post-operative symptom scores

Patient	Pruritus score		Xanthoma score	
	Preoperative	Post-operative	Preoperative	Post-operative
1	4	1	0	0
2	4	1	0	0
3	2	1	4	3

Pruritus score: 0, none; 1, rubbing or mild scratching when undistracted; 2, active scratching without evident skin abrasions; 3, abrasions evident; 4, cutaneous mutilation, hemorrhage, and scarring evident. Xanthoma score: 0, none; 1, minimal, <20 scattered individual lesions; 2, moderate, >20 lesions not interfering with or limiting activities; 3, disfiguring, large numbers of lesions causing distortion of the face or extremities; 4, disabling, large numbers of lesions interfering with walking or hand use because of excess size and number.

There were no post-operative complications in two of the patients. One patient was brought back to the operating room on post-operative day 1 for evacuation of hematoma, with no active bleeding found. The same patient’s post-operative course was complicated by cholangitis which was successfully treated with antibiotics. This patient went on to have progressive liver failure, ultimately requiring liver transplant ~1 year after biliary diversion. Repeat liver biopsy at the time of biliary diversion revealed Stage F3 fibrosis. Transplant was uncomplicated and successful to date.

Mean preoperative total/direct bilirubin was 7.0/ 5.4 mg/dl. Mean preoperative cholesterol was 774 mg/dl. Mean preoperative bile acid was 331 μmol/l. Post-operative lab values can be seen in Table 3. All patients had improvement in their pruritus and/or xanthomas scores. The patient that progressed to transplant did have recurrence of Stage 4 xanthomas and Stage 2 pruritus Table 2.

**Table 3:** Pertinent preoperative and post-operative laboratory values

Patient	Total/direct bilirubin (mg/dl)		Total cholesterol (mg/dl)		Bile acids (μmol/l)	
	Preoperative	Post-operative	Preoperative	Post-operative	Preoperative	Post-operative
1	1.5/1.1	3.2/2.1	310	Not recorded	281	11
2	2.9/2.1	2.6/2.1	425	289	196	76
3	16.5/13.0	18.7/13.2	1588	1018	517	528

## DISCUSSION

The creation of a cholecystocolostomy has been shown to be effective in patients with PFIC, but to our knowledge, this is the first series using this technique in patients with AGS [9]. The goal of this procedure is to improve symptoms in patients with medically refractory AGS without the problems associated with external drainage and the morbidity and mortality associated with transplantation. This series suggests that cholecystocolostomy is an effective surgical option for patients with AGS with severe symptoms despite optimal medical therapy. All three patients experienced improvement in their pruritus score. In addition, the patient who had a significant xanthoma burden also experienced improvement with a 1-point reduction in their symptom score. This technique can be considered as an option prior to liver transplant.

This technique has several advantages to existing surgical options for AGS. From a surgical perspective, it is technically feasible procedure and involves a single colonic anastomosis and the cholecystocolostomy. The gallbladder fundus is easily accessible without the need for significant dissection. It is also a large target for the single layer cholecystocolostomy. The patient who underwent an entirely open cholecystocolostomy had previous abdominal surgery, including a gastrostomy tube and open liver biopsy. Although two of the three patients underwent laparoscopic-assisted procedures, we believe that this technique could be done entirely laparoscopically. This is further supported by the study performed by Daio *et al*. Their series of 20 patients with PFIC who underwent laparoscopic cholecystocolostomy had no major morbidity or mortality. The laparoscopic technique was also effective, with normalization of bile acids at 12 months in 85% of the patients [9]. Cholecystocolostomy eliminates the need for any external drainage system or stoma creation, which can be burdensome to patients and their caregivers due to its cosmetic and physiologic challenges.

It is unclear why one of the patients had further liver failure and ultimately required transplant, though it is likely related to irreversible advanced liver disease. Although they initially improved symptomatically, their laboratory values continued to worsen, and their symptoms returned. This patient underwent ERCP via the roux limb ~9 months after cholecystocolostomy and prior to transplant. Bile was visualized in the roux limb, but contrast was only visualized in the pancreatic duct. There was no filling of the intrahepatic ducts. This patient was also treated with antibiotics for cholangitis after biliary diversion. It is possible that this also contributed to the failure of the procedure.

There are several limitations to this study, the most important of which is that it is a retrospective review of three patients with medically refractory AGS. Though the series is small, AGS is a rare disorder and patients typically require complex multidisciplinary subspecialty tertiary care. While it may be difficult to draw definitive conclusions, this study provides foundation for a larger multi-institution study that would provide more insight into surgical interventions for AGS.

In summary, we believe cholecystocolostomy to be a safe and effective technique for biliary drainage in medically refractory AGS. Cholecystocolostomy improves patient symptoms without physiologic derangement and can be performed using minimally invasive techniques. It should be considered prior to transplantation when evaluating surgical options for patients with AGS and symptoms refractory to medical management.

## References

[1] Balistreri WF . Intrahepatic cholestasis. J Pediatr Gastroenterol Nutr 2002;35:S17–23. 12151816 10.1097/00005176-200207001-00006

[2] Turnpenny PD , Ellar S. Alagille syndrome: pathogenesis, diagnosis and management. Eur J Hum Genet 2012;20:251–7. 21934706 10.1038/ejhg.2011.181 PMC3283172

[3] Krantz ID , Piccoli DA, Spinner NB. Alagille syndrome. J Med Genet 1997;34:152–7. 9039994 10.1136/jmg.34.2.152 PMC1050871

[4] Whitington PF , Whiting GL. Partial external diversion of bile for the treatment of intractable pruritus associated with intrahepatic cholestasis. Gastroenterology 1988;95:130–6. 3371608 10.1016/0016-5085(88)90301-0

[5] Emerick KM , Whitington PF. Partial external biliary diversion for intractable pruritus and xanthomas in Alagille syndrome. Hepatology 2002;35:1501–6. 12029636 10.1053/jhep.2002.33332

[6] Ng VL , Ryckman FC, Porta G, Miura IK, de Carvalho E, Servidoni MF, et al. Long-term outcome after partial external biliary diversion for intractable pruritus in patients with intrahepatic cholestasis. J Pediatr Gastroenterol Nutr 2000;30:152–6. 10697133 10.1097/00005176-200002000-00011

[7] Mattei P , von Allmen D, Piccoli D, Rand E. Relief of intractable pruritus in Alagille syndrome by partial external biliary diversion. J Pediatr Surg 2006;41:104–7. 16410117 10.1016/j.jpedsurg.2005.10.014

[8] Modi BP , Suh MY, Jonas MM, Lillehei C, Kim HB. Ileal exclusion for refractory symptomatic cholestasis in Alagille syndrome. J Pediatr Surg 2007;42:800–5. 17502187 10.1016/j.jpedsurg.2006.12.032

[9] Diao M , Li L, Zhang JS, Ye M, Cheng W. Laparoscopic cholecystocolostomy: a novel surgical approach for the treatment of progressive familial intrahepatic cholestasis. Ann Surg 2013;258:1028–33. 23187749 10.1097/SLA.0b013e31827905eb

